# Correlates of Protection for M Protein-Based Vaccines against Group A Streptococcus

**DOI:** 10.1155/2015/167089

**Published:** 2015-05-25

**Authors:** Shu Ki Tsoi, Pierre R. Smeesters, Hannah R. C. Frost, Paul Licciardi, Andrew C. Steer

**Affiliations:** ^1^Group A Streptococcus Research Group, Murdoch Childrens Research Institute, Melbourne, VIC 3052, Australia; ^2^Centre for International Child Health, University of Melbourne, Melbourne, VIC 3052, Australia; ^3^Pneumococcal Research Group, Murdoch Childrens Research Institute, Melbourne, VIC 3052, Australia; ^4^Department of Paediatrics, Royal Children's Hospital, Melbourne, VIC 3052, Australia

## Abstract

Group A streptococcus (GAS) is known to cause a broad spectrum of illness, from pharyngitis and impetigo, to autoimmune sequelae such as rheumatic heart disease, and invasive diseases. It is a significant cause of infectious disease morbidity and mortality worldwide, but no efficacious vaccine is currently available. Progress in GAS vaccine development has been hindered by a number of obstacles, including a lack of standardization in immunoassays and the need to define human correlates of protection. In this review, we have examined the current immunoassays used in both GAS and other organisms, and explored the various challenges in their implementation in order to propose potential future directions to identify a correlate of protection and facilitate the development of M protein-based vaccines, which are currently the main GAS vaccine candidates.

## 1. Introduction

Group A streptococcus (GAS, otherwise known as* Streptococcus pyogenes*) is one of the most important causes of infectious disease morbidity and mortality, with an estimated prevalence of severe disease of at least 18.1 million cases leading to more than 500,000 deaths annually [1]. Much of the burden exists in less developed countries where control strategies are difficult to implement and ensure efficacy. A vaccine is therefore widely acknowledged as an important strategy to reduce the burden of GAS disease globally. Several vaccine candidates are presently in various stages of preclinical development [2], and a limited number have also reached early phase clinical trials [3, 4]. A key element in vaccine development is the availability of a validated and standardized immunoassay that correlates with immune protection. However, despite decades of research, there is no single standardized immunoassay and certainly no human correlate of protection (CoP) established for GAS [5]. In the case of other bacterial pathogens, such as* Streptococcus pneumoniae* and group B streptococcus (GBS), for which vaccines are widely available or development is underway, CoPs provide a means of assessing the true efficacy and immunogenicity of potential vaccines [6, 7].

## 2. Immune Response to GAS Infection

A solid understanding of bacterial pathogenesis and host immune response lays the foundations for robust CoPs. It is widely agreed that the M protein is an important virulence factor of GAS, conferring both adhesion and antiphagocytic properties through binding of various host proteins and interacting with the complement pathway [8, 9]. The M protein is an alpha-helical coiled-coil dimer extending from the surface of the bacteria as a fibril [10]. Its structure is divided into conserved, central variable, and N-terminal hypervariable regions [11]. Some M proteins may have a nonhelical portion at the distal end of the N-terminal region, but the significance of this is unknown [9]. There are a number of A, B, C, and D repeats that vary between the different M proteins, with increasing sequence conservation downstream of the hypervariable region.

The current GAS classification system, known as* emm*-typing, is based on sequencing of 10–15% of the* emm* gene (which encodes the M protein) with 223* emm*-types identified worldwide [12, 13]. Recently, a new adjunct method of classification, known as the “*emm*-cluster system” has been developed, which organizes the 223* emm*-types into 48 distinct* emm*-clusters based on the phylogenetic analysis of the whole M protein and its structural and binding properties [14–16].

The main immune mechanisms against GAS are thought to be antibody-mediated. Studies in mice have demonstrated that mucosal IgA antibodies against the M protein prevent colonization and adherence of GAS [17, 18]. Binding of type-specific IgG antibodies to the N-terminus of the M protein in the host activates the complement pathway, leading to deposition of C3b and facilitating phagocytosis and killing [18]. Limited evidence suggests that these type-specific antibodies provide long-lasting protection against homologous strains [19]. Whilst the conserved region of the M protein has not been as extensively studied as the N-terminus, it has also been shown to elicit osonophagocytic antibodies [20–23]. Antibodies produced against the B repeat region of the M protein are not thought to be opsonic and thus unlikely to be protective [24].

Memory B cells and T cells also appear to play a role in propagating immunity [21], and several immunodominant T cell epitopes have been mapped [18], although the exact mechanisms of cell-mediated immunity in GAS are still unclear [21, 23]. Non-M protein virulence factors such as C5a peptidase, lipoteichoic acid, protein F, and* Streptococcal* fibronectin-binding protein have been shown in animal studies to raise protective antibodies that contribute to host immunity, particularly in preventing colonization [18, 25]. Antibodies to streptococcal toxins such as streptococcal pyrogenic exotoxins A, B, and C and streptococcal erythrogenic exotoxin B have also been proposed to play a role in* in vivo* immunity [18]. Other potentially immunogenic antigens that have been investigated as vaccine candidates include streptococcal protective antigen, serum opacity factor, streptococcal pili,* S. pyogenes* cell envelope protease, and GAS carbohydrate [25]. Whilst all of the above may play a role in GAS immunity, it is believed that opsonic type-specific antibodies remain responsible for clearing infection [18].

**Figure 1:**
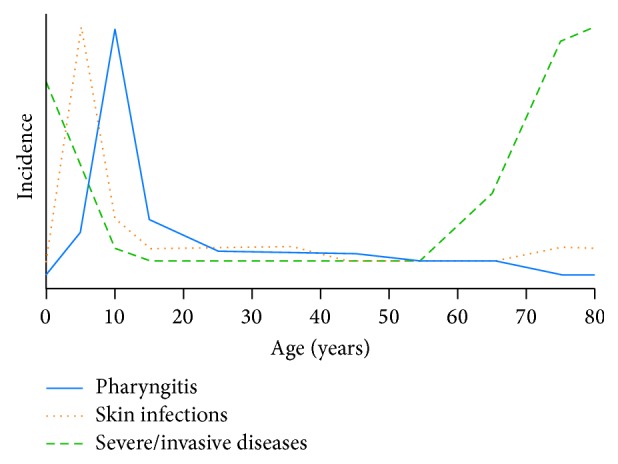
Schematic representation of incidence of group A streptococcal diseases by age using data from epidemiological reports [27, 30–33].

The exact natural history of immunity against GAS infections also remains unclear, although there are distinct peaks of increased incidence at different age groups for each GAS disease (Figure 1). For instance, the incidence of GAS superficial infections is high in children but decreases in adulthood [20]. Anti-GAS antibody levels in adults are much higher compared with children, suggesting that this natural acquisition of antibodies due to exposure over time provides protective immunity against GAS diseases later in life [20, 26]. Similarly for severe and invasive GAS diseases, there is decreased incidence in adulthood; the significant peak in the elderly is likely to be due to comorbid illness and immunosenescence [27–29].

## 3. Immune Correlates of Protection

### 3.1. Definitions

**Figure 2:**
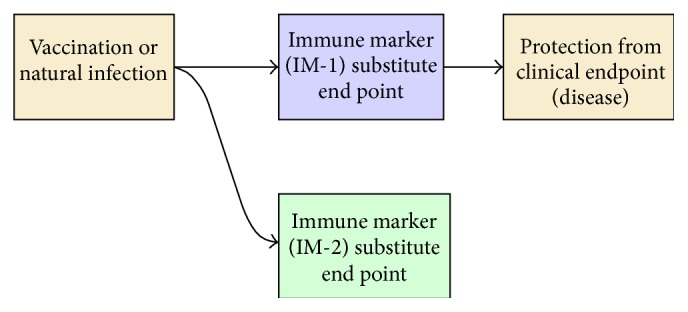
Process of immunity and correlates of protection. Immune markers 1 and 2 (IM-1, IM-2) are correlates of protection, but only IM-1 is a surrogate. Arrows imply direct causal relationships. Figure adapted from WHO [34].

A “correlate” is “an attribute that is statistically associated with an endpoint (without the association necessarily being causal),” whilst a “surrogate” or “mechanistic CoP” (mCoP) has the added criterion of being part of the causal pathway and the mechanism by which a vaccine induces protection (Figure 2) [34].

### 3.2. Rationale for Establishing Correlates of Protection in GAS

In 2011, it was agreed that a roadmap for GAS vaccine development was needed to harness the efforts of the international community and two key components were identified as (1) definition of human CoPs for GAS and (2) development of high throughput standardized assays that accurately represent* in vivo* immunity [5, 25, 35].

A key long-term advantage of CoPs is in enabling vaccine licensure by obviating the need for demonstrating field efficacy in the scenario where efficacy trials are logistically or ethically challenging [36]. Establishing the efficacy and safety of vaccine candidates can be a lengthy and expensive endeavour involving large-scale phase III trials. Whilst it is likely that the first GAS vaccine candidate to be licensed will be approved based on phase III trials using pharyngitis as an end-point, CoPs may provide an alternative for second generation vaccines. If an immunological marker is established as a CoP and it is demonstrated that a vaccine candidate meets or exceeds an immune threshold, then it may be approved based on serological data alone [34]. Successful precedents include vaccines for meningococcal serogroups B [37] and C [38–40], influenza [41],* S. pneumonia* [42], and* Haemophilus influenzae* type b (Hib) [43]. Not only can CoPs fast-track the licensure process and potentially reduce costs, but they can also be useful when placebo randomized controlled trials (RCT) are no longer possible or ethical (as in the case of Hib) [34]. This may be the case if a successful GAS vaccine is developed; it may be unethical to allow cases of RHD and potentially fatal invasive diseases to occur in subsequent vaccine development trials when a vaccine would prevent them.

Correlates of protection may also be used to inform seroepidemiology studies and surveillance of populations postvaccination. This could be relevant to GAS because of high global diversity of strains that may evoke different patterns of immune responses and to evaluate whether a vaccine will introduce pressure that leads to the emergence of new strains. Further applications that could be explored include passive protection, where the CoP is used to determine the amount of antibody to administer in passive immunizations and as screening or diagnostic tools, for instance, in testing antibody levels against rubella for pregnant women [34].

Thus, establishing a CoP would provide an adjunct tool of great benefit in GAS vaccine development [6]. However, numerous challenges have hindered CoP development for GAS. Firstly, there are technical difficulties in developing a high quality, standardized immunoassay for GAS. Further, the utilization of the assay to determine a CoP for GAS raises its own set of challenges in terms of study design and use of animal or human models.

## 4. Development of a Standardized Immunoassay for GAS

The optimal immunoassay used to define CoPs should ideally meet several criteria. It should be reproducible across different laboratories with a common standard operating protocol and equivalent data sets. For GAS, one of the key points will be ensuring that antigens and GAS strains used by different laboratories are comparable. The methodology needs to be based on a thorough understanding of GAS pathogenesis, host interactions, and immune response in order to be clinically relevant [36]. A practical assay should have a high throughput.

It is worth noting that these criteria for the establishment of a robust standardized immunoassay in GAS are important not only in terms of defining a CoP, but also in securing reliable immunological data for the conventional aspects of vaccine development to enable proof-of-concept studies, successful progression through trial phases, and ensuring ongoing funding from the relevant sources. A robust immunoassay will also be particularly critical in helping to overcome the specific challenges in GAS vaccine development such as ensuring coverage across the many different strains and variation in geographical distribution.

There are currently two main types of immunoassays utilized in GAS research: assays that give a purely quantitative measurement of antibody such as the enzyme-linked immunosorbent assay (ELISA) and functional bactericidal assays that demonstrate immunity through evidence of phagocytic killing of GAS (Table 1). Assays that have been established for other pathogens that have potential application to GAS include those for* S. pneumoniae* and GBS [6]. The opsonophagocytic assays proposed as CoPs may not necessarily apply to all GAS protective antigens.

### 4.1. Enzyme-Linked Immunosorbent Assay

The ELISA has been used for quantification of antibodies in GAS in the development of all the major GAS vaccine candidates in testing of both rabbit and mice antisera, as well as human antisera in two phase I trials [4, 17, 21, 23, 44–49]. The ELISA has been used successfully in* S. pneumoniae* research to establish CoPs [6] and in GBS to propose potential CoPs values [7], suggesting that the ELISA may be a good candidate for determining GAS CoPs.

One issue is the choice of antigen to use, such as recombinant M protein [17], or synthetic peptides [4, 44, 47]. The peptide method is generally favoured in preclinical studies as it allows for the testing of antibodies specific to the vaccine epitope only and minimizes the amount of non-specific antibodies detected [10, 44, 47]. However, there is a theoretical concern that the folding structure of peptides may be altered from the normal structure when within a whole M protein and consequently give results that may not be clinically relevant [50].

There are potential methods to increase specificity for ELISAs using the full M protein. For example, in* S. pneumoniae* research, initial ELISAs were found to correlate poorly with* in vivo* reactions due to measurement of nonfunctional antibodies to pneumococcal cell wall polysaccharide (C-PS) in addition to anti-capsular antibodies [51]. This problem was overcome by preabsorption of serum with highly purified C-PS before use in the ELISA, to remove non-functional antibodies [51]. Similarly, preabsorption with pneumococcal type 22F capsular polysaccharide neutralized antibodies cross-reactive to common epitopes, leaving only serotype-specific antibodies to be measured in the ELISA [52]. For GAS, the addition of a preabsorption step with an unrelated M peptide or full M protein could increase the specificity and detection of functional antibodies. A slightly different approach was taken in GBS, where alterations to the antigen were made [53, 54]. The GBS III polysaccharide was mixed with methylated human serum albumin (HSA) to increase adherence of the antigen to the microtitre plates and coupled with biotin or nonmethylated HAS [53], and a subsequent study found using nonmethylated HSA produced the highest specificity and sensitivity [55]. In GAS, mixing the full M protein with fibrinogen or HSA to bind the B repeat and C repeat portions of the M protein, respectively, could reduce detection of non-functional antibodies.

A high quality ELISA should also be reproducible and standardized across laboratories. There is currently no standardized protocol between GAS laboratories, making it difficult to confidently compare results between studies. Two approaches could potentially be used to ameliorate this issue: (1) attempting to regulate all steps and reagents used or (2) providing a standard serum for laboratories to use as a reference. The latter is utilized for* S. pneumoniae*, where ELISAs are standardized against the reference serum, 007 sp, created from volunteers immunized with the 23-valent vaccine [58]. This allows for the continued comparison of assay results in vaccine evaluations.

### 4.2. Multiplexed Flow Cytometry Assays

Martins et al. took a novel approach by developing a multiplexed fluorescent immunoassay using Luminex Multi-Analyte Profiling technology (Luminex Corp., Austin, TX) [59]. IgG antibody responses against nine common GAS antigens (including the M protein) conjugated to fluorescent microspheres were simultaneously measured using either human sera or animal antibodies. Whilst the assay quantified antibody responses to antigens in a high throughput fashion, a high degree of nonspecific binding of IgG to microspheres was reported and no comparison with ELISA or bactericidal assays was performed.

### 4.3. Bactericidal Assays

The principle of GAS bactericidal assays is to incubate whole blood with bacterial inoculum and then determine bactericidal activity by measuring colony-forming units (CFU). There are two variations, the direct bactericidal test (DBT) and the indirect bactericidal test (IBT). The DBT utilizes the test subject's whole blood as a source for serum and phagocytes. The IBT only uses serum from the test subject and nonimmune human blood from a separate donor as the source of phagocytes, with the aim of reducing variability as donors are screened to ensure that there are no type-specific or cross-reactive antibodies present [56]. This also allows for testing of animal sera [56]. The IBT has lower sensitivity with low antibody titres, leading to the development of an optional preopsonization step where antisera is first incubated with GAS to increase antibody binding before adding the source of phagocytes [56]. Currently, the IBT is the most commonly used method to assess functional immunity to GAS [4, 44–48]. Despite this, there are still many variations in methodology between laboratories (Table 2), and even in the World Health Organization established protocols, two main methods of performing the IBT are described [56].

Inter-assay variability is high because of the different methodologies and because of the unstandardized element of donor blood. The need for fresh human blood is problematic as it means that assays need to be performed within two hours of venepuncture. Both the donor and test subject must have the same blood group, or only group O blood can be used, to prevent an ABO incompatibility reaction [56]. The IBT is labour intensive and low throughput, making it a less suitable choice for determining a CoP for GAS.

A variation of the IBT using light microscopy of Wright smear stains to define opsonization rather than counting CFU has also been used to test GAS vaccine candidates [46, 60]. However, in the phase I trial of the hexavalent GAS vaccine, less of a response was seen in the IBT when compared to this version of the opsonophagocytic assay [46], suggesting that in some cases association of bacteria with the cell wall of leukocytes may not equate to killing.

### 4.4. HL-60 Opsonophagocytic Assay

The HL-60 opsonophagocytic assay (OPA) is an alternative functional opsonic assay that could potentially be utilized in GAS, given its prior success with* S. pneumonia* [61] and GBS [62]. The HL-60 cells used as the source of phagocytes are human promyelocytic leukaemia cells capable of being cultured and differentiated, thus eliminating the need for human donors and potentially decreasing inter-assay variability. The assay tests the bactericidal activity and ability of sera to opsonize viable bacteria in the presence of differentiated HL-60 neutrophils and complement.

### 4.5. *Streptococcus pneumoniae* HL-60 OPA

Romero-Steiner et al. first developed the HL-60 OPA [61] to measure functional antibodies against* S. pneumoniae*. They demonstrated for seven serotypes that the HL-60 OPA correlated highly with OPAs using peripheral blood leukocytes (PBL), the previous standard for effector cells [61]. Over time, there have been a number of variations trialled and modifications made to the assay (Table 3).

The HL-60 OPA was tested in various multilaboratory trials using multiplexing and automated colony counting, showing robust inter-assay and interlaboratory reproducibility and validation [63, 64], and a standardized protocol is now available online [65]. However, it was found that the execution of the OPA is not always tightly regulated across all laboratories, and whilst overall agreement is sufficient, there remains higher variability compared to ELISA [66]. Further, the correlation between ELISA, HL-60, and PBL OPAs is poor for some specific pneumococcal serotypes. For example, five serum samples tested for serotype 19F immunity had elevated ELISA IgG titres but no opsonophagocytic activity [61]. This may in fact be a problem with the ELISA; a study comparing ELISA and OPA in children with human immunodeficiency virus (HIV) in South Africa found raised ELISA antibody titres in both infected and noninfected groups, but lower OPA titres in HIV-infected individuals, suggesting that the ELISA could be detecting non-protective antibodies [67].

Nevertheless, evidence of bactericidal activity of functional antibodies is desired when considering licensure of a new vaccine candidate, and thus the WHO has determined for* S. pneumoniae* conjugate vaccines that an OPA titre of 1 : 8 in conjunction with an ELISA antibody concentration of at least 0.35 *μ*g/mL demonstrates that a candidate provides sufficient protection to be considered for licensure [66].

It has been proposed that the use of the 007sp reference serum could also increase standardization [66]. The development of a reference serum for GAS would create a degree of uniformity across laboratories and help facilitate immunoassay and vaccine development. Furthermore, the establishment of a standard collection of GAS isolates for laboratories to reference would increase standardization in the performance of immunoassays used in vaccine studies.

### 4.6. Group B Streptococcus HL-60 OPA

The HL-60 OPA was adapted for use in GBS by validating it against the previous “gold standard” OPA that uses human PBLs [62]. Attempts to increase throughput of the assay have focussed on developing a fluorescent OPA (flOPA) using fluorescence activated cell sorting (FACS) to measure the uptake of fluorescently labelled, whole killed GBS cells, or antigen-coated fluorescent microspheres [68]. The flOPA has multiple advantages including the ability to be multiplexed, the ability to standardize complement and effector cells, and a faster running time [69]. A criticism of previous flOPAs has been the inability to distinguish between adhered and internalized bacteria [68]. Fabbrini et al. have overcome this for GBS with pHrodo, a pH-sensitive fluorogenic dye that only emits a bright red signal in an acidic environment [68]. This pH-dependent reaction means that pHrodo-labelled bacteria are only detected when they are in phagolysosomes. Bacteria that are bound to the HL-60 cell wall show only a very low signal on FACS, and thus the actual bactericidal antibodies can be measured rather than just measuring adhered antibodies. This method was shown to be reproducible and as sensitive and specific as a traditional OPA [68]. The potential applications of this technique are promising; however no studies using human sera have been carried out to date, and FACS remains an expensive method requiring skilled technicians.

## 5. Implementing Immunoassays to Determine a Correlate of Protection

Once a standardized immunoassay is established, studies can be carefully designed to determine what levels of immune markers correlate with protection.

### 5.1. Geographic Variation

A key challenge in establishing a CoP is the inherent heterogeneity present within populations and environments. There is a distinct variance in distribution of strains in developed countries when compared with resource poor regions such as Africa and the Pacific [70, 71]. The highest rates of GAS diseases are in these low socioeconomic areas [1, 72], where crowding, poor hygiene, and tropical climate are likely to result in greater exposure and infection pressures. These differences may cause varying protective antibody levels for each population. Early studies suggested that different GAS strains require different levels of antibodies to be considered immune [19]. If that is the case, it may be difficult to accurately determine specific CoPs due to the challenges of obtaining comprehensive data from developing regions and the apparently constantly evolving epidemiological landscape [1, 70].

### 5.2. Host Factor Variation

Individual host factors can also alter CoPs, as a person's immunity changes with age and between individuals. The level of antibody that is protective in a young healthy adult may not protect against infection in an elderly individual [34]. GAS bactericidal antibodies have been shown to persist for up to 30 years but to varying degrees between persons [19], making it potentially difficult to predict CoPs in the long-term. Cell-mediated immunity involving B and T cells has also been shown to play a role in long-term memory. In particular, memory B cells are capable of mounting a rapid response to GAS infection in animal studies [21], potentially meaning that long-term immunity may be more dependent on an individual's cellular response and that low circulating antibody titres do not necessarily correlate with a lack of protection. For instance, the Hib conjugate vaccine was licensed based on a CoP of serum antibody concentration of 0.04–1.0 *μ*g/mL but variations from this range have been shown in different studies to be protective, and it is thought that immunologic memory and population differences have not been accounted for [43].

### 5.3. Vaccine Composition and Mode of Delivery

The choice of antigen(s) for a vaccine candidate, route of administration, and immune marker targeted can also result in different CoPs. For instance, the protective titre required for type-specific antibodies raised by the 30-valent vaccine [44] may be different from that raised by the J8-DT vaccine against the conserved region of the M protein [22]. Secretory IgA antibodies are part of mucosal protection and inhibit GAS colonization but may not always necessarily correlate with serum IgG levels or protection against invasive disease [17]. There is some evidence that mucosal IgA antibodies may be able to contribute to immunity in certain cases, with intranasal immunisation of mice shown to induce salivary antibodies and protect against subsequent intranasal challenge [82, 83]. These studies raise the possibility that mucosally delivered vaccines (e.g., intranasal) would require separate development of mucosal assays and CoPs [36]. It is also unclear whether serum opsonophagocytic IgG antibodies involved in clearing infection are protective against recurrent skin infections, as most studies of immune protection against GAS have traditionally focused on pharyngitis.

### 5.4. Animal Models

Animal models provide a way of studying* in vivo* immunity and pathogenesis, and pathogens that have used animal models to determine CoPs include* Clostridium tetani* and* Clostridium botulinum* [34]. However, there have been difficulties in developing accurate disease models and bridging the gap from animal to human immune response because GAS is a human-only pathogen [84]. There have been numerous animal models of GAS infections developed, spanning from murine models for virulence and vaccine studies to RHD in rats; invasive disease in rabbits or pigs; and pharyngitis in primates [85]. Murine models have been used in studies of several vaccine candidates [47, 86]. However, there are several limitations to these models: only a select number of GAS strains are virulent in mice; repeated passage of strains may be needed but result in mutations altering virulence; colonization is often difficult to achieve; and true pharyngitis does not occur [85]. As such, murine models are unlikely to give an accurate indication of human CoPs for GAS. Nonhuman primate models have been established in an attempt to provide a more clinically relevant model of GAS pharyngitis [87]. Although there is variable reproducibility of the severity of clinical disease seen in monkeys, they have been shown to produce type-specific opsonic antibodies (measured by bactericidal assays) [87] and could be utilized in challenge or vaccine studies to evaluate CoPs.

### 5.5. Study Design

Traditionally, CoPs have been established through a RCT phase III vaccine trial as has been the case for Hib, tuberculosis, and* S. pneumoniae* vaccine candidates [34, 88]. Other studies nested within RCTs may also be used to inform CoPs, as demonstrated by a case-control study of the acellular pertussis vaccine [89]. The lack of phase III trials has hindered the establishment of CoPs for GAS as RCTs ideally allow for an understanding of the relationships between the vaccine, immune markers, and clinical endpoint (Figure 2) for CoPs to be determined with appropriate statistical power.

Similar challenges face the GBS vaccine field where there is no vaccine beyond phase II trials currently available. Lin et al. used a case-control study to estimate a CoP in order to facilitate vaccine licensure [7]. Maternal and cord serum samples from infants across 14 hospitals were compared by ELISA to establish a relationship between IgG levels against GBS and early-onset disease in neonates. In this study, an antibody titre ≥5 *μ*g/mL was protective against neonatal disease [7]. A case-control study conducted by Baker et al. also evaluated maternal antibody levels required to protect against GBS early-onset disease, finding that an overall titre ≥1 *μ*g/mL conferred a 70% risk reduction [90]. More specifically, it was identified that different antibody levels were found to be protective for the different GBS types Ia, III, and V [90]. There are limited data from human natural infection studies for a number of GAS vaccine candidates. For example, a study of serum IgG antibodies against p145, the parent peptide of J8, showed an age-related increase in antibody titres suggesting that these antibodies may correlate with protection over the life course (Figure 1) [20]. Similarly, anti-C5a peptidase titres were found to be in significantly higher concentrations in adults than in children in Minnesota [91]. Further, a study of children aged 5–14 years in Mexico observed higher titres of anti-GAS carbohydrate antibodies that correlated with reduced colonization of the pharynx suggesting that there may be a threshold protective antibody titre [92]. These studies suggest that defining a CoP without data from phase III clinical trials will be challenging, although an important consideration in the future remains. Additionally, whilst this evidence is insufficient to establish a CoP, it may help to inform clinical “proof-of-concept,” that is, determination of whether an intervention is biologically active or inactive [93]. Proof-of-concept is an important bridging step in ensuring that government bodies, investors, and pharmaceutical companies continue to support the vaccine development process and allow for larger scale efficacy studies where a CoP may then be established.

Data from phase I and II clinical trials are also relevant to the current status of GAS vaccine research as has been the case for phase II trials of the botulinum F toxoid vaccine [97] and the Meningococcal C conjugate vaccine in the United Kingdom [40]. In the case of Meningococcal C, protective titres extrapolated from animal studies were used to inform human CoPs, and vaccine licensure was approved by comparing CoPs with the preexisting vaccine [34].

### 5.6. Human Challenge Model

Given the limited clinical applicability of animal models and the potential drawbacks of phase III clinical trials, human challenge models would provide a controlled method for testing of new vaccines. Many pathogens including, but not limited to* S. pneumoniae* [98], cholera [99], malaria [100], and dengue virus [101], utilize human challenge models in their fields. Three pharyngeal challenge studies of GAS were carried out in the 1970s with a total of 178 volunteers (Table 4) [94–96]. Importantly, the trials were safe, with penicillin administered within the therapeutic timeframe and patients closely monitored to prevent progression into invasive disease or autoimmune sequelae. In a potential GAS human challenge model, individuals would be infected with a carefully predetermined virulent strain of GAS known to cause pharyngitis and followed through to see if they develop clinical disease. By analyzing blood samples taken at different time points and comparing immune markers with clinical outcomes, one could then establish potential CoPs.

## Figures and Tables

**Table 1:** Advantages and disadvantages of different immunoassays currently utilized in GAS research.

Immunoassay	Advantages	Disadvantages
ELISA	High-throughput	May measure non-functional antibodies
	Easily standardized	
	Reproducible	

Bactericidal assays	Measures functional antibodies	Labour-intensive
		Inter-assay variability
		Use of whole human blood imposes two hour time restriction
		No controls for DBT

**Table 2:** Variations across methods used in performing the indirect bactericidal test.

Assay component	Variations	Reference(s)
Bacteria growth phase	Stationary	[56]
	(Mid)logarithmic	[44–47]

Bacterial dilution	10^−4^ with 1 : 4 serial dilution	[56]
	10^−4^ with 1 : 7 serial dilution	[56]
	10^−5^ with 1 : 4 serial dilution	[46, 47, 57]

Growth media	Todd-Hewitt broth only	[44–47]
	Todd-Hewitt broth + 20% normal calf/horse serum	[56]
	Serum broth	[57]
	Addition of 1% neopeptone	[56]

Serum	Heat inactivation	[47, 57]

**Table 3:** Various modifications attempted with the *Streptococcus pneumoniae* HL-60 OPA.

Modification	Details	Advantages	Disadvantages	Reference(s)
Baby rabbit complement (BRC) 3-4 weeks	The use of BRC was compared with human complement for six serotypes and no significant difference found	BRC is easier to obtain Less variability between donors Does not require screening for type-specific antibodies		[61]

Radiolabelled or fluorescence-labelled bacteria	Bacteria were radiolabelled or fluorescence-labelled and then flow cytometry analysis used to automatically quantify phagocytosis	Increases throughput of assay	Poor sensitivity in infants with low antibody concentrations Flow cytometers are expensive, not readily available in all laboratories Radioactive waste	[73–76]

Testing multiple serotypes simultaneously	Pneumococci serotypes were modified to be resistant to a different antibiotic each. Strains were tested as one bacterial sample then plated on selective media. Twofold, fourfold and sevenfold multispecificity OPAs were performed	Decreases amount of serum and other materials required Increases throughput of assay	Theoretical concern of increasing antibiotic resistance and creating a “superbug,” although the antibiotics chosen for the assay are not used clinically	[77–79]

Automated colony counting	Colonies were stained with 2,3,5-triphenyl tetrazolium chloride dye in an agar overlay for contrast and then counted with an automatic colony counter	Increases throughput of assay (reduces counting time from hours to 2-3 minutes) Increases accuracy by visualizing colonies <0.2 mm in diameter Increases accuracy by visualizing colonies <0.2 mm in diameter Increases accuracy by visualizing colonies <0.2 mm in diameter Increases accuracy by visualizing colonies <0.2 mm in diameter		[80, 81]

**Table 4:** Previous GAS human challenge studies. CFU/mL: colony-forming units per millilitre.

Year	Strain	Dose (CFU/mL)	Number of participants	Number of not vaccinated	Clinical illness in nonvaccine group	Reference
1971	M1	4 × 10^6^	50	25	52%	[94]
1973	M1	4 × 10^6^	44	23	74%	[95]
1975	M3, M12	5 × 10^6^	84	36	44%	[96]
